# Vanillin Activates Human Bitter Taste Receptors TAS2R14, TAS2R20, and TAS2R39

**DOI:** 10.3389/fnut.2021.683627

**Published:** 2021-07-09

**Authors:** Gabriella Morini, Marcel Winnig, Timo Vennegeerts, Gigliola Borgonovo, Angela Bassoli

**Affiliations:** ^1^University of Gastronomic Sciences, Pollenzo, Italy; ^2^IMAX Discovery GmbH, Dortmund, Germany; ^3^Axxam S.p.A. Bresso, Italy; ^4^DeFENS – Department of Food, Environmental and Nutritional Sciences, University of Milan, Milan, Italy

**Keywords:** chemoreception, vanillin, bitter taste, TAS2Rs taste receptors, biosensor, sensory nutrition

## Abstract

Vanilla is widely used in food preparation worldwide for its sensory properties, mainly related to its fragrance, being vanillin the major compound present in the processed vanilla. Vanillin is also known to elicit bitterness as a secondary sensory sensation, but the molecular mechanism of its bitterness has never been reported. Assay buffers of vanillin were tested *in vitro* on all known 25 human bitter taste receptors TAS2Rs. Three receptors, TAS2R14, TAS2R20, and TAS2R39, were activated, showing that these receptors are mediating the bitterness of vanillin. The result could be useful to improve the overall sensory profile of this broadly used food ingredient, but even more could represent the starting point for further studies to investigate the potential of vanillin in sensory nutrition and other pharmaceutical applications.

## Introduction

Vanilla is probably the world's most popular flavoring material, with extensive applications in food, beverages, cosmetics, perfumery, and pharmaceuticals. The source of vanilla is the bean, or pod, of the tropical orchids of the genus Vanilla (principally *Vanilla planifolia Andrews*, also known as *Vanilla fragrans* –Salisbury- Ames). Vanilla beans have no vanilla flavor upon harvesting, and acquire it only after curing, during which vanillin β-D-glucoside and related β-D-glucosides come into contact with β-D-glucosidases, with the result that free vanillin (4-hydroxy-3-methoxybenzaldehyde) and related substances are released (1). Quantitatively, vanillin is the major compound present in the processed vanilla pods, usually ca. 2.0–2.5%, while the number of minor components is around 200. Vanillin is also the main component of non-synthetic vanilla flavors obtained by biotechnological processes (2), products gaining importance in the food industry due to increasing consumer demand for non-synthetic food additives (3). Vanilla was also used in traditional medicine in Mesoamerica, where it originally comes from, for many diseases including those of the respiratory and gastrointestinal tracts (4), and was listed with the same indication in the American Pharmacopeia until 1916 (5). Most of the earlier medicinal uses of vanilla have given way to studies on its functional uses. Vanillin has shown to have anticancer activity and to regulate inflammatory response and oxidative stress (6, 7), and to be a powerful antimicrobial (8), also suitable to innovative application as in arthroplasty implants (9). Recently vanillin has proven to have appetite-enhancing effect (10) and to improve the gut microbiota composition on mice (11).

Vanillin is utilized for its iconic and unmistakable flavor, often described as “sweet.” It is mainly used in favorites like ice cream, candies, cakes, and cookies; vanilla aroma is also known to enhance the perceived sweetness (12). Despite the common association with sweetness, vanillin is also reported for its capability to elicit bitterness as a secondary sensory sensation (13, 14). The bitterness of vanillin may be a limitation for its use at certain concentrations, and recommendations are sometimes given on commercial preparations to avoid overdosing, which could bring to undesired bitterness. Whereas, the sensory features of vanillin are well-known to food technologist, the molecular mechanism of bitterness of this compound has never been studied, and the bitter taste receptors responsible of this biological activity are not reported in literature and specialized databases as the BitterDB (15).

The identification of specific chemoreceptors for taste was followed by the observation that they are widely expressed throughout the body (16), where they mediate “systemic” responses of different nature, ranging from innate immunity to the release of hormones involved in appetite control, through various specialized mechanisms (17). It has also become apparent that taste receptors and their polymorphisms are associated with several human disorders (18). This has opened new perspectives on the function and evolutionary role of these receptors (19), and given rise to the possibility of using them as targets for personalized health interventions (20). It has to be noted that many of the compounds tested on taste receptors are not of natural source, due to the difficulty of isolating, identify and obtain them in fair amount, while the number of easily available synthetic compounds is very high (many drugs). Therefore, it is nowadays a fundamental step to investigate the biological properties of taste active food components to unravel their possible role and applications in health promoting function mediated by the activation of taste receptors. To understand which bitter taste receptor(s) is/are involved in the perception of a widely used compound such as vanillin is therefore of great relevance. In our study we performed *in vitro* assays of vanillin on all 25 known human TAS2Rs bitter taste receptors, and we identified TAS2R14, TAS2R20, and TAS2R39 to be the receptors mediating the bitterness of vanillin.

## Materials and Methods

### Preparation of Vanillin and Reference Compounds

Vanillin (Sigma Aldrich) was dissolved at the indicated concentrations using assay buffer (130 mM NaCl, 5 mM KCl, 1 mM MgCl_2_, 2 mM CaCl_2_, 5 mM NaHCO_3_, and 20 mM HEPES, pH 7.4). Vanillin solutions were freshly prepared on the day of the experiment. For the biological tests, several compounds were used as reference TAS2R agonists, purchased from different suppliers (Sigma Aldrich, Santa Cruz, Tocris Bioscience) and dissolved in assay buffer. The complete list of compounds used as TAS2R references is reported in Table 1. We found that these receptor/ligand combinations produce reliable, robust responses. Pairs for TAS2R1, TAS2R7, TAS2R10, and TAS2R20 were identified by us (not published). The remaining receptor/ligand pairs are published in Meyerhof et al. (21).

**Table 1:** List of reference compounds used as agonists for the human TAS2R receptors *in vitro* assays.

**Receptor**	**Compound**	**Concentration**
TAS2R1	Oxyphenonium bromide	3 mM
TAS2R3	Chloroquine	10 mM
TAS2R4	Colchicine	1 mM
TAS2R5	1,10-phenanthroline	600 μM
TAS2R7	Aurintrincarboxylic acid	30 μM
TAS2R8	Chloramphenicol	300 μM
TAS2R9	Ofloxacin	3 mM
TAS2R10	Oxyphenonium bromide	3 mM
TAS2R13	Denatonium benzoate	3 mM
TAS2R14	Aristolochic acid	30 μM
TAS2R16	Salicin	3 mM
TAS2R20	Ritanserin	30 μM
TAS2R30	Amarogentin	10 μM
TAS2R31	Aristolochic acid	30 μM
TAS2R38	Sinigrin	3 mM
TAS2R39	Denatonium benzoate	3 mM
TAS2R40	Cinchonine	100 μM
TAS2R43	Aristolochic acid	30 μM
TAS2R46	Strychnine	10 μM
TAS2R50	Andrographolide	30 μM

*The reported concentrations are the highest used for each receptor*.

### Vectors and cDNAs, Cell Culture, and Transfection

All human TAS2R cDNAs were cloned into a pcDNA5/FRT expression vector (Thermo Fisher Scientific) 3′ of the first 45 amino acids of the rat somatostatin receptor 3 (21). The cDNA for the chimeric G-protein alpha subunit was cloned into a pcDNA6 expression vector (Thermo Fisher Scientific). Amino acid sequences of all 25 TAS2Rs and the chimeric G-protein are contained in Supplementary Table 1.

All experiments were performed using HEK293 PEAKrapid cells (ATCC, CRL-2828). This line was derived from the human embryonic kidney line, HEK 293, by stable transfection with a plasmid encoding a temperature-sensitive mutant of the SV40 large T antigen. The cells are very efficiently transfectable with DNA. They support the replication of recombinant plasmids with the Epstein-Barr virus (EBV) oriP or SV40 origin of replication and transiently maintain a high copy number, which can greatly increase the amount of recombinant protein that can be produced from the cells.

Cells were cultured in high glucose DMEM media with stable L-glutamine (ECM0103L Euroclone) plus 10% (v/v) FBS (ECS0180L Euroclone) and 1% (v/v) Pen/Strep 10K/10K stock (17-602E Lonza). A stable cell line was produced by constitutively expressing the chimeric G protein Gα16i/o44 alpha subunit. The cDNAs for the different TAS2Rs were transiently transfected using Lipofectamine2000 (Thermo Fisher Scientific) according to the manufacturer's instructions. Cells were plated at a density of 20,000 cells/well in 384 plates (GR-4332 Twin Helix srl) and analyzed 24 h after transfection.

### Calcium Imaging Analysis and Quantification

For the calcium imaging experiments, 2 μM Cal520-AM calcium-sensitive dye (AAT Bioquest) and 2.5 mM probenecid (Molecular Probes), an inhibitor of organic anion transport, were diluted in assay buffer. Twenty-four hours after transfection, cells were loaded with Cal520-AM and probenecid solution and incubated 3 h at room temperature. After incubation, cells were washed once with the assay buffer before data acquisition.

Calcium responses of transfected cells upon test compound application were measured using a Fluorometric Imaging PlateReader, FLIPR Tetra, (Molecular Devices). The acquisition was performed by starting 10 s before compound injection and acquiring one image every second for the first minute, and one image every 5 s for the following 30 s. Calcium kinetics were visualized using Molecular Devices Screenworks 4.1. For the calculation of dose-response curves, responses were calculated as the difference between maximal and minimal Relative Fluorescence Unit (RFU) values in a selected time window (11–60 s) and were normalized to basal well-fluorescence (timepoint 1, before compound injection) in order to compensate for differences in cell density (ΔF/F0). All the results are averages of at least four replicates from two independent experiments. Mock-transfected cells (cells transfected with empty plasmid used as negative control) were always measured in parallel on the same plates using the same compound concentrations used to examine the cells expressing the various TAS2Rs.

All calculations and plots were made using Microsoft Excel 365 and GraphPad Prism 8.0.

## Results

In order to identify the taste receptor(s) mediating the bitterness of vanillin, we transiently expressed all 25 known human TAS2Rs individually in HEK293 PEAKrapid cells, constitutively expressing the chimeric G protein Gα16i/o44 alpha subunit, which couples to the bitter receptors and triggers the intracellular calcium mobilization, measured as the readout in the assay (Supplementary Figure 1).

**Figure 1:**
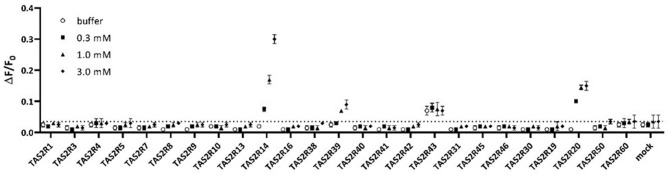
Assay buffer (white circles) or 0.3mM vanillin (black filled squares), 1mM vanillin (black-filled triangles), and 3mM vanillin (black filled diamonds) tested on all human TAS2Rs. Only TAS2R14, TAS2R20, and TAS2R39 showed concentration-dependent specific activation. Data are plotted as ΔF/F0 above time, where ΔF/F0 are RFU normalized for the basal fluorescence at time zero.

Assay buffer or vanillin at 0.3, 1, and 3 mM were tested on all known human TAS2Rs. Among all 25 TAS2Rs three receptors, TAS2R14, TAS2R20, and TAS2R39, were activated in these experimental conditions, as shown in Figure 1.

The response of TAS2R43 to vanillin is not significantly different than that to buffer (baseline response, white circles). Therefore, we concluded that TAS2R43 does not respond to Vanillin. TAS2R43 always shows higher baseline responses, which might be caused by constitutive activity. The result indicates that TAS2R14, TAS2R20, and TAS2R39 are the bitter taste receptors mediating the bitterness of vanillin.

**Figure 2:**
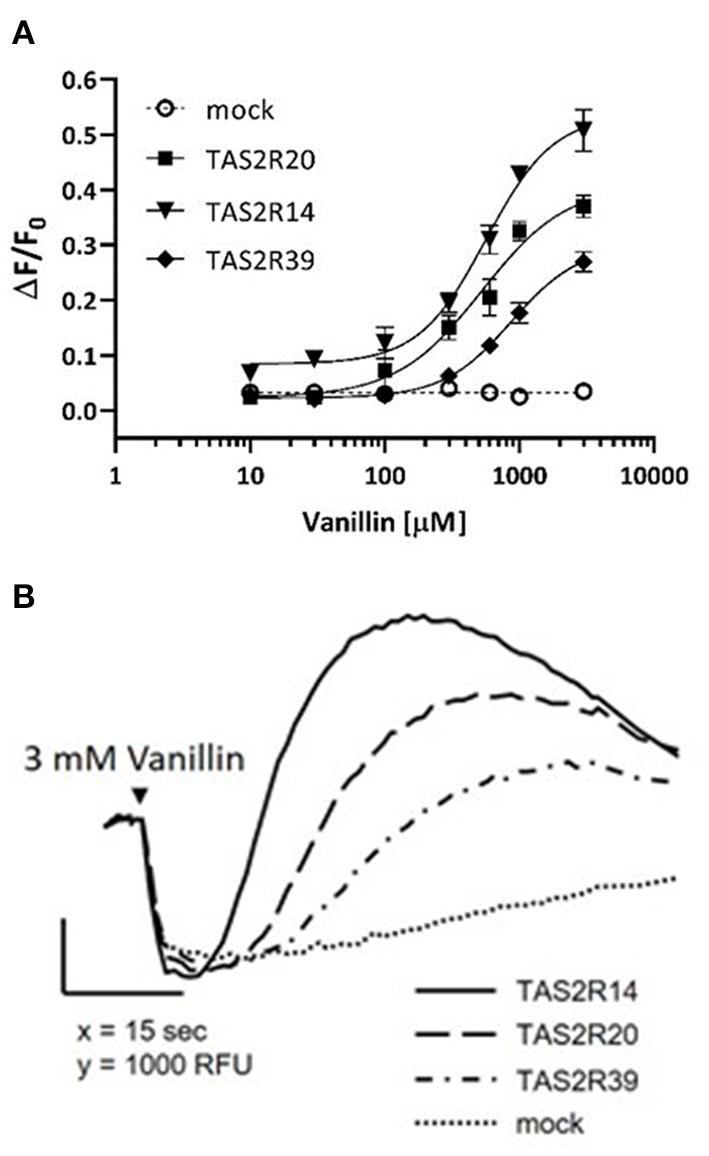
Vanillin activity on human TAS2R14, TAS2R20, and TAS2R39 bitter taste receptors. **(A)** The figure shows dose-response curves obtained after stimulation of HEK293 PEAKrapid Gα16Gi/o44 cells overexpressing TAS2R14 (black filled triangles), TAS2R20 (black filled squares), and TAS2R39 (black filled diamonds). Responses of mock-transfected cells (empty plasmid) are indicated by the dotted line. Error bars represent the standard deviation. Experimental data are the full points, curve fitting is represented by the solid line. **(B)** Calcium-responses of TAS2R14 (solid line), TAS2R20 (dashed line), and TAS2R39 (dash-dotted line) expressing cells to 3mM vanillin. Receptor-expressing cells were loaded with the calcium indicator and fluorescence emissions recorded before and after exposure of the cells to vanillin. Responses of mock-transfected cells to the same concentration of vanillin is shown as negative control (dotted line).

Dose-response curves were obtained after stimulation of HEK293 PEAKrapid Gα16Gi/o44 cells overexpressing TAS2R14, TAS2R20, and TAS2R39 bitter receptors (Figure 2).

Each point represents the mean of four replicates. Functional assays revealed that vanillin activation of all three TAS2Rs was concentration dependent, with an EC_50_-value of 0.57 ± 0.11 mM for TAS2R14, 0.53 ± 0.09 mM for TAS2R20 and 0.87 ± 0.19 mM for TAS2R39. Notably, vanillin showed less potency and efficacy on all three receptors compared to the reference agonists used herein (Supplementary Figure 2).

## Discussion

Human TAS2R20 (previously indicated as TAS2R49 or TAS2R56) (22), has been defined a narrowly tuned receptor, TAS2R39 an intermediately tuned and TAS2R14 a broadly tuned bitter taste receptor (23).

The discovery that taste receptors are widely expressed extraorally (16), inscribe them into the broad chemosensory system which recognizes exogenous (plants or processed food) and endogenous (products of metabolism or microbiota) agonists (24). TAS2R14 and TAS2R20 are among the most expressed TAS2Rs in normal (non-pathological) extraoral tissues (25). In the gastrointestinal tract, bitter taste receptors activation has been demonstrated to initially stimulate the release of ghrelin, (a hormone promoting food intake), then that of cholecystokinin (CCK) able to reduce gastric motility and to induce satiety, and of glucagon-like peptide 1 (GLP-1), a hormone that contribute to the release of insulin and to the reduction of glucagon in the pancreas, inducing satiety and glucose absorption (17, 26). Plants traditionally used to prevent or treat type 2 diabetes are very often bitter (27), and for some of them, their activity through TAS2Rs has been proven (28, 29). The discovery that vanillin, one of the most common food flavoring agent, activates TAS2R14 and TAS2R20, bitter taste receptors expressed in the intestine (26), will be a starting point to study its possible bioactivity as modulator of gastrointestinal functions, which could lead to the decrease in energy intake and the reduction of glucose variations after meals in humans.

Bitter taste receptors activation in the lung have been established to relax airway smooth muscle, eliciting a bronchodilatory function (30). Moreover, the bitter compounds inducing bronchodilatory effect present the advantage to show little tachyphylaxis (partial or total loss of the efficacy over time) compared to β-agonists (31). Therefore, agonists of the bitter taste receptors may have interesting applications in asthma therapeutics (32), even if the bitter taste of the drug may represent a limitation to its use (33). The fact that vanillin has a familiar and generally pleasing aroma would represent a great advantage in such applications.

The immune system, also referred as the “sixth sense” being able to detect pathogens in the body like the other senses detect stimuli in the external environment (34, 35), has been shown to use some of the same chemosensory receptors (24, 36). TAS2R38, abundantly expressed in the respiratory tract, is able to respond to some quorum-sensing molecules secreted by some pathogens, and its activation stimulates the production of NO, with biocidal activity (37). Following this discovery, the same bitter taste receptor has been proven to be broadly tuned for bacterial quorum sensing molecules (38). TAS2R38 is well-known to present two main global high-frequency *TAS2R38* haplotypes in worldwide populations, the PTC “taster” PAV (encoding proline, alanine, and valine at the respective variant sites) and the PTC “non-taster” AVI (encoding alanine, valine, and isoleucine at these sites) (39, 40). The AVI allele does not respond even to quorum sensing molecules (37): this represents a limitation in the use of TAS2R38 as a target of antibacterial drugs, since the high frequency AVI haplotype will not respond to them.

Therefore, other bitter taste receptor, among which TAS2R14 and TAS2R20, which in the present work we have discovered to be activated by vanillin, have been identified to respond to several bacterial acyl homoserine lactones (41) and quinolones (42), making them potential therapeutic target for upper respiratory infections treatments, particularly in TAS2R38 AVI/AVI individuals (43).

In the gastrointestinal tract the role of bitter taste receptors in innate immunity has not been established, but a detailed study of TAS2R38 variants in Africa (39) demonstrated that their distribution is not correlated to diet, raising the possibility that common haplotypes are under selection due to non-dietary biological processes, being the interaction with the microbiota one of the most likely. Therefore, the fact that vanillin is able to activate bitter taste receptors expressed in the gastrointestinal tract, could represent a starting point to study its possible role in shaping the gut microbiota.

In our work we identified TAS2R14, TAS2R20, and TAS2R39 to be the receptors mediating the bitterness of vanillin. This outcome could be useful to improve the overall sensory profile of this broadly used food ingredient. But even more intriguing, since TAS2R14 and TASR20 are broadly expressed in extraoral tissues, and have been identified as receptors of particular relevance for the physiological actions of bitter compounds beyond taste, vanillin, being volatile, very pleasant in flavor and GRAS-listed (Generally Recognized As Safe), represents an interesting compound to be tested for pharmaceutical and sensory nutrition applications mediated by these bitter taste receptors.

## Data Availability Statement

The original contributions presented in the study are included in the article/Supplementary Material, further inquiries can be directed to the corresponding author/s.

## Author Contributions

GM, MW, and AB: conceptualization and writing—original draft preparation. GM, MW, AB, TV, and GB: methodology and writing—review and editing. MW, TV, and GB: formal analysis. All authors have read and agreed to the published version of the manuscript.

## Conflict of Interest

MW and TV are full-time employees of IMAX Discovery GmbH, Dortmund, Germany, and Axxam S.p.A. Bresso (MI), Italy. The remaining authors declare that the research was conducted in the absence of any commercial or financial relationships that could be construed as a potential conflict of interest.
