# Supplementary material for: Vanillin Activates Human Bitter Taste Receptors TAS2R14, TAS2R20, and TAS2R39
Source: Front Nutr. 2021 Jul 9;8:683627. doi: 10.3389/fnut.2021.683627 (PMC8298857; doi:10.3389/fnut.2021.683627)
Supplement: Supplementary file 1 [file Data_Sheet_1.docx]

Vanillin Activates Human Bitter Taste Receptors TAS2R14, TAS2R20 and TAS2R39

Gabriella Morini ^1^*, Marcel Winnig ^2,3^, Timo Vennegeerts ^2,3^, Gigliola Borgonovo ^4^ and Angela Bassoli ^4^

^1^ University of Gastronomic Sciences, Pollenzo, Bra (CN), Italy

^2^ IMAX Discovery GmbH, Dortmund, Germany

^3^ Axxam S.p.A. Bresso (MI), Italy

^4^ DeFENS – Department of Food, Environmental and Nutritional Sciences, University of Milan, Milan, Italy

Supplementary Material

**Supplementary Figure 1: Original calcium-responses of TAS2Rs to reference agonists.** Receptor-expressing cells were loaded with the calcium indicator and fluorescence emissions recorded before and after exposure of the cells to the bitter substances as indicated in table 1 (solid lines). Responses of mock-transfected cells to the same concentration of the substances are shown as negative control (dashed lines).


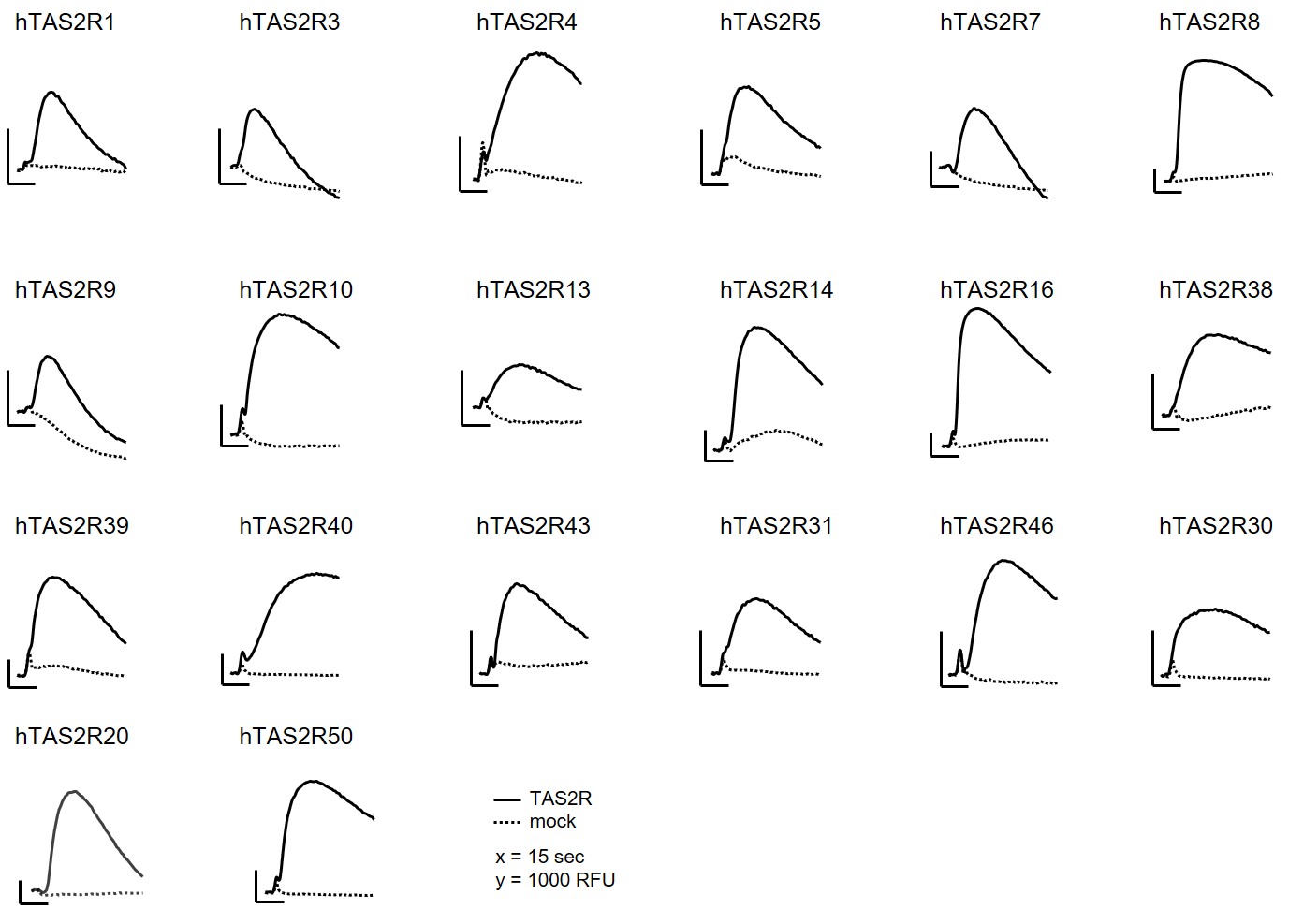


**Supplementary Figure 2: Activation of TAS2R14, TAS2R20 and TAS2R39 by their reference agonists and vanillin.** (A) Concentration-dependent activation of TAS2R14 by aristolochic acid (white squares) and vanillin (white circles). EC_50_-values are 0.65 ± 0.09 μM for aristolochic acid and 0.57 ± 0.11 mM for vanillin. (B) Concentration-dependent activation of TAS2R20 by ritanserin (white squares) and vanillin (white circles). EC_50_-values are 5.4 ± 0.9 μM for ritanserin and 0.53 ± 0.09 mM for vanillin. (C) Concentration-dependent activation of TAS2R39 by denatonium benzoate (white squares) and vanillin (white circles). EC_50_-values are 87 ± 11 μM for denatonium benzoate and 0.87 ± 0.19 mM for vanillin. Activation of mock-transfected cells to the highest concentration of vanillin (black filled circles) and the reference substances (black filled squares) are shown as negative controls.


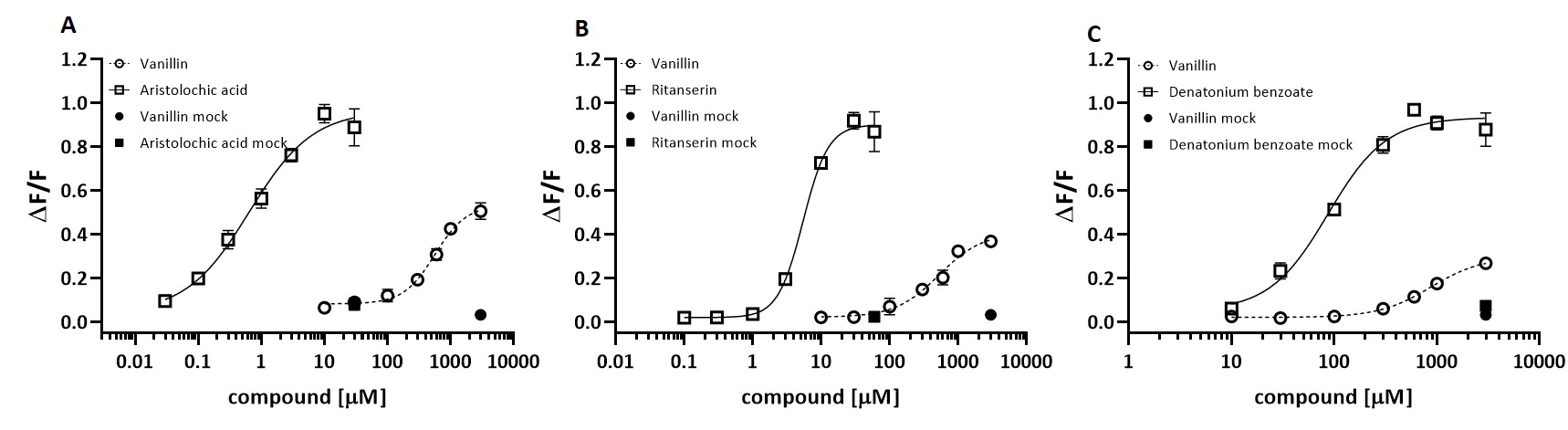


**Supplemental table 1:** Amino acid sequences of the chimeric G-protein and the TAS2Rs used in the present study.

**Gα16i/o44**

MARSLTWRCCPWCLTEDEKAAARVDQEINRILLEQKKQDRGELKLLLLGPGESGKSTFIKQMRIIHGAGYSEEERKGFRPLVYQNIFVSMRAMIEAMERLQIPFSRPESKHHASLVMSQDPYKVTTFEKRYAAAMQWLWRDAGIRAYYERRREFHLLDSAVYYLSHLERITEEGYVPTAQDVLRSRMPTTGINEYCFSVQKTNLRIVDVGGQKSERKKWIHCFENVIALIYLASLSEYDQCLEENNQENRMKESLALFGTILELPWFKSTSVILFLNKTDILEEKIPTSHLATYFPSFQGPKQDAEAAKRFILDMYTRMYTGCVDGPEGSNLKKEDKEIYCHMTCATDTNNIQVVFDAVTDIIIANNLKDCGLF

**TAS2R1**

1 MLESHLIIYF LLAVIQFLLG IFTNGIIVVV NGIDLIKHRK MAPLDLLLSC

51 LAVSRIFLQL FIFYVNVIVI FFIEFIMCSA NCAILLFINE LELWLATWLG

101 VFYCAKVASV RHPLFIWLKM RISKLVPWMI LGSLLYVSMI CVFHSKYAGF

151 MVPYFLRKFF SQNATIQKED TLAIQIFSFV AEFSVPLLIF LFAVLLLIFS

201 LGRHTRQMRN TVAGSRVPGR GAPISALLSI LSFLILYFSH CMIKVFLSSL

251 KFHIRRFIFL FFILVIGIYP SGHSLILILG NPKLKQNAKK FLLHSKCCQ

**TAS2R3**

1 MMGLTEGVFL ILSGTQFTLG ILVNCFIELV NGSSWFKTKR MSLSDFIITT

51 LALLRIILLC IILTDSFLIE FSPNTHDSGI IMQIIDVSWT FTNHLSIWLA

101 TCLGVLYCLK IASFSHPTFL WLKWRVSRVM VWMLLGALLL SCGSTASLIN

151 EFKLYSVFRG IEATRNVTEH FRKKRSEYYL IHVLGTLWYL PPLIVSLASY

201 SLLIFSLGRH TRQMLQNGTS SRDPTTEAHK RAIRIILSFF FLFLLYFLAF

251 LIASFGNFLP KTKMAKMIGE VMTMFYPAGH SFILILGNSK LKQTFVVMLR

301 CESGHLKPGS KGPIFS

**TAS2R4**

1 MLRLFYFSAI IASVILNFVG IIMNLFITVV NCKTWVKSHR ISSSDRILFS

51 LGITRFLMLG LFLVNTIYFV SSNTERSVYL SAFFVLCFMF LDSSSVWFVT

101 LLNILYCVKI TNFQHSVFLL LKRNISPKIP RLLLACVLIS AFTTCLYITL

151 SQASPFPELV TTRNNTSFNI SEGILSLVVS LVLSSSLQFI INVTSASLLI

201 HSLRRHIQKM QKNATGFWNP QTEAHVGAMK LMVYFLILYI PYSVATLVQY

251 LPFYAGMDMG TKSICLIFAT LYSPGHSVLI IITHPKLKTT AKKILCFKK

**TAS2R5**

1 MLSAGLGLLM LVAVVEFLIG LIGNGSLVVW SFREWIRKFN WSSYNLIILG

51 LAGCRFLLQW LIILDLSLFP LFQSSRWLRY LSIFWVLVSQ ASLWFATFLS

101 VFYCKKITTF DRPAYLWLKQ RAYNLSLWCL LGYFIINLLL TVQIGLTFYH

151 PPQGNSSIRY PFESWQYLYA FQLNSGSYLP LVVFLVSSGM LIVSLYTHHK

201 KMKVHSAGRR DVRAKAHITA LKSLGCFLLL HLVYIMASPF SITSKTYPPD

251 LTSVFIWETL MAAYPSLHSL ILIMGIPRVK QTCQKILWKT VCARRCWGP

**TAS2R7**

1 MADKVQTTLL FLAVGEFSVG ILGNAFIGLV NCMDWVKKRK IASIDLILTS

51 LAISRICLLC VILLDCFILV LYPDVYATGK EMRIIDFFWT LTNHLSIWFA

101 TCLSIYYFFK IGNFFHPLFL WMKWRIDRVI SWILLGCVVL SVFISLPATE

151 NLNADFRFCV KAKRKTNLTW SCRVNKTQHA STKLFLNLAT LLPFCVCLMS

201 FFLLILSLRR HIRRMQLSAT GCRDPSTEAH VRALKAVISF LLLFIAYYLS

251 FLIATSSYFM PETELAVIFG ESIALIYPSS HSFILILGNN KLRHASLKVI

301 WKVMSILKGR KFQQHKQI

**TAS2R8**

1 MFSPADNIFI ILITGEFILG ILGNGYIALV NWIDWIKKKK ISTVDYILTN

51 LVIARICLIS VMVVNGIVIV LNPDVYTKNK QQIVIFTFWT FANYLNMWIT

101 TCLNVFYFLK IASSSHPLFL WLKWKIDMVV HWILLGCFAI SLLVSLIAAI

151 VLSCDYRFHA IAKHKRNITE MFHVSKIPYF EPLTLFNLFA IVPFIVSLIS

201 FFLLVRSLWR HTKQIKLYAT GSRDPSTEVH VRAIKTMTSF IFFFFLYYIS

251 SILMTFSYLM TKYKLAVEFG EIAAILYPLG HSLILIVLNN KLRQTFVRML

301 TCRKIACMI

**TAS2R9**

1 MPSAIEAIYI ILIAGELTIG IWGNGFIVLV NCIDWLKRRD ISLIDIILIS

51 LAISRICLLC VISLDGFFML LFPGTYGNSV LVSIVNVVWT FANNSSLWFT

101 SCLSIFYLLK IANISHPFFF WLKLKINKVM LAILLGSFLI SLIISVPKND

151 DMWYHLFKVS HEENITWKFK VSKIPGTFKQ LTLNLGAMVP FILCLISFFL

201 LLFSLVRHTK QIRLHATGFR DPSTEAHMRA IKAVIIFLLL LIVYYPVFLV

251 MTSSALIPQG KLVLMIGDIV TVIFPSSHSF ILIMGNSKLR EAFLKMLRFV

301 KCFLRRRKPF VP

**TAS2R10**

1 MLRVVEGIFI FVVVSESVFG VLGNGFIGLV NCIDCAKNKL STIGFILTGL

51 AISRIFLIWI IITDGFIQIF SPNIYASGNL IEYISYFWVI GNQSSMWFAT

101 SLSIFYFLKI ANFSNYIFLW LKSRTNMVLP FMIVFLLISS LLNFAYIAKI

151 LNDYKMKNDT VWDLNMYKSE YFIKQILLNL GVIFFFTLSL ITCIFLIISL

201 WRHNRQMQSN VTGLRDSNTE AHVKAMKVLI SFIILFILYF IGMAIEISCF

251 TVRENKLLLM FGMTTTAIYP WGHSFILILG NSKLKQASLR VLQQLKCCEK

301 RKNLRVT

**TAS2R13**

1 MESALPSIFT LVIIAEFIIG NLSNGFIVLI NCIDWVSKRE LSSVDKLLII

51 LAISRIGLIW EILVSWFLAL HYLAIFVSGT GLRIMIFSWI VSNHFNLWLA

101 TIFSIFYLLK IASFSSPAFL YLKWRVNKVI LMILLGTLVF LFLNLIQINM

151 HIKDWLDRYE RNTTWNFSMS DFETFSVSVK FTMTMFSLTP FTVAFISFLL

201 LIFSLQKHLQ KMQLNYKGHR DPRTKVHTNA LKIVISFLLF YASFFLCVLI

251 SWISELYQST VIYMLCETIG VFSPSSHSFL LILGNAKLRQ AFLLVAAKVW

301 AKR

**TAS2R14**

1 MGGVIKSIFT FVLIVEFIIG NLGNSFIALV NCIDWVKGRK ISSVDRILTA

51 LAISRISLVW LIFGSWCVSV FFPALFATEK MFRMLTNIWT VINHFSVWLA

101 TGLGTFYFLK IANFSNSIFL YLKWRVKKVV LVLLLVTSVF LFLNIALINI

151 HINASINGYR RNKTCSSDSS NFTRFSSLIV LTSTVFIFIP FTLSLAMFLL

201 LIFSMWKHRK KMQHTVKISG DASTKAHRGV KSVITFFLLY AIFSLSFFIS

251 VWTSERLEEN LIILSQVMGM AYPSCHSCVL ILGNKKLRQA SLSVLLWLRY

301 MFKDGEPSGH KEFRESS

**TAS2R16**

1 MIPIQLTVFF MIIYVLESLT IIVQSSLIVA VLGREWLQVR RLMPVDMILI

51 SLGISRFCLQ WASMLNNFCS YFNLNYVLCN LTITWEFFNI LTFWLNSLLT

101 VFYCIKVSSF THHIFLWLRW RILRLFPWIL LGSLMITCVT IIPSAIGNYI

151 QIQLLTMEHL PRNSTVTDKL ENFHQYQFQA HTVALVIPFI LFLASTIFLM

201 ASLTKQIQHH STGHCNPSMK AHFTALRSLA VLFIVFTSYF LTILITIIGT

251 LFDKRCWLWV WEAFVYAFIL MHSTSLMLSS PTLKRILKGK C

**TAS2R38**

1 MLTLTRIRTV SYEVRSTFLF ISVLEFAVGF LTNAFVFLVN FWDVVKRQPL

51 SNSDCVLLCL SISRLFLHGL LFLSAIQLTH FQKLSEPLNH SYQAIIMLWM

101 IANQANLWLA ACLSLLYCSK LIRFSHTFLI CLASWVSRKI SQMLLGIILC

151 SCICTVLCVW CFFSRPHFTV TTVLFMNNNT RLNWQIKDLN LFYSFLFCYL

201 WSVPPFLLFL VSSGMLTVSL GRHMRTMKVY TRNSRDPSLE AHIKALKSLV

251 SFFCFFVISS CAAFISVPLL ILWRDKIGVM VCVGIMAACP SGHAAVLISG

301 NAKLRRAVMT ILLWAQSSLK VRADHKADSR TLC

**TAS2R39**

1 MLGRCFPPDT KEKQQLRMTK LCDPAESELS PFLITLILAV LLAEYLIGII

51 ANGFIMAIHA AEWVQNKAVS TSGRILVFLS VSRIALQSLM MLEITISSTS

101 LSFYSEDAVY YAFKISFIFL NFCSLWFAAW LSFFYFVKIA NFSYPLFLKL

151 RWRITGLIPW LLWLSVFISF SHSMFCINIC TVYCNNSFPI HSSNSTKKTY

201 LSEINVVGLA FFFNLGIVTP LIMFILTATL LILSLKRHTL HMGSNATGSN

251 DPSMEAHMGA IKAISYFLIL YIFNAVALFI YLSNMFDINS LWNNLCQIIM

301 AAYPASHSIL LIQDNPGLRR AWKRLQLRLH LYPKEWTL

**TAS2R40**

1 MATVNTDATD KDISKFKVTF TLVVSGIECI TGILGSGFIT AIYGAEWARG

51 KTLPTGDRIM LMLSFSRLLL QIWMMLENIF SLLFRIVYNQ NSVYILFKVI

101 TVFLNHSNLW FAAWLKVFYC LRIANFNHPL FFLMKRKIIV LMPWLLRLSV

151 LVSLSFSFPL SRDVFNVYVN SSIPIPSSNS TEKKYFSETN MVNLVFFYNM

201 GIFVPLIMFI LAATLLILSL KRHTLHMGSN ATGSRDPSMK AHIGAIKATS

251 YFLILYIFNA IALFLSTSNI FDTYSSWNIL CKIIMAAYPA GHSVQLILGN

301 PGLRRAWKRF QHQVPLYLKG QTL

**TAS2R41**

1 MQAALTAFFV LLFSLLSLLG IAANGFIVLV LGREWLRYGR LLPLDMILIS

51 LGASRFCLQL VGTVHNFYYS AQKVEYSGGL GRQFFHLHWH FLNSATFWFC

101 SWLSVLFCVK IANITHSTFL WLKWRFLGWV PWLLLGSVLI SFIITLLFFW

151 VNYPVYQEFL IRKFSGNMTY KWNTRIETYY FPSLKLVIWS IPFSVFLVSI

201 MLLINSLRRH TQRMQHNGHS LQDPSTQAHT RALKSLISFL ILYALSFLSL

251 IIDAAKFISM QNDFYWPWQI AVYLCISVHP FILIFSNLKL RSVFSQLLLL

301 ARGFWVA

**TAS2R42**

1 MATELDKIFL ILAIAEFIIS MLGNVFIGLV NCSEGIKNQK VFSADFILTC

51 LAISTIGQLL VILFDSFLVG LASHLYTTYR LGKTVIMLWH MTNHLTTWLA

101 TCLSIFYFFK IAHFPHSLFL WLRWRMNGMI VMLLILSLFL LIFDSLVLEI

151 FIDISLNIID KSNLTLYLDE SKTLFDKLSI LKTLLSLTSF IPFSLSLTSL

201 LFLFLSLVRH TRNLKLSSLG SRDSSTEAHR RAMKMVMSFL FLFIVHFFSL

251 QVANWIFFML WNNKYIKFVM LALNAFPSCH SFILILGNSK LRQTAVRLLW

301 HLRNYTKTPN ALPL

**TAS2R43**

1 MITFLPIIFS SLVVVTFVIG NFANGFIALV NSIESFKRQK ISFADQILTA

51 LAVSRVGLLW VLLLNWYSTV LNPAFNSVEV RTTAYNIWAV INHFSNWLAT

101 TLSIFYLLKI ANFSNFIFLH LKRRVKSVIL VMLLGPLLFL ACHLFVINMN

151 EIVRTKEFEG NMTWKIKLKS AMYFSNMTVT MVANLVPFTL TLLSFMLLIC

201 SLCKHLKKMQ LRGKGSQDPS TKVHIKALQT VISFLLLCAI YFLSIMISVW

251 SFGSLENKPV FMFCKAIRFS YPSIHPFILI WGNKKLKQTF LSVFWQMRYW

301 VKGEKTSSP

**TAS2R31**

1 MTTFIPIIFS SVVVVLFVIG NFANGFIALV NSIERVKRQK ISFADQILTA

51 LAVSRVGLLW VLLLNWYSTV FNPAFYSVEV RTTAYNVWAV TGHFSNWLAT

101 SLSIFYLLKI ANFSNLIFLH LKRRVKSVIL VMLLGPLLFL ACQLFVINMK

151 EIVRTKEYEG NMTWKIKLRS AVYLSDATVT TLGNLVPFTL TLLCFLLLIC

201 SLCKHLKKMQ LHGKGSQDPS TKVHIKALQT VIFFLLLCAV YFLSIMISVW

251 SFGSLENKPV FMFCKAIRFS YPSIHPFILI WGNKKLKQTF LSVLRQVRYW

301 VKGEKPSSP

**TAS2R45**

1 MITFLPIIFS ILVVVTFVIG NFANGFIALV NSTEWVKRQK ISFADQIVTA

51 LAVSRVGLLW VLLLNWYSTV LNPAFYSVEL RTTAYNIWAV TGHFSNWPAT

101 SLSIFYLLKI ANFSNLIFLR LKRRVKSVIL VVLLGPLLFL ACHLFVVNMN

151 QIVWTKEYEG NMTWKIKLRR AMYLSDTTVT MLANLVPFTV TLISFLLLVC

201 SLCKHLKKMQ LHGKGSQDPS TKVHIKVLQT VISFFLLCAI YFVSVIISVW

251 SFKNLENKPV FMFCQAIGFS CSSAHPFILI WGNKKLKQTY LSVLWQMRYW

301 VKGEKPSSP

**TAS2R46**

1 MITFLPIIFS ILIVVTFVIG NFANGFIALV NSIEWFKRQK ISFADQILTA

51 LAVSRVGLLW VLVLNWYATE LNPAFNSIEV RITAYNVWAV INHFSNWLAT

101 SLSIFYLLKI ANFSNLIFLH LKRRVKSVVL VILLGPLLFL VCHLFVINMN

151 QIIWTKEYEG NMTWKIKLRS AMYLSNTTVT ILANLVPFTL TLISFLLLIC

201 SLCKHLKKMQ LHGKGSQDPS MKVHIKALQT VTSFLLLCAI YFLSIIMSVW

251 SFESLENKPV FMFCEAIAFS YPSTHPFILI WGNKKLKQTF LSVLWHVRYW

301 VKGEKPSSS

**TAS2R30**

1 MITFLPIIFS ILIVVIFVIG NFANGFIALV NSIEWVKRQK ISFVDQILTA

51 LAVSRVGLLW VLLLHWYATQ LNPAFYSVEV RITAYNVWAV TNHFSSWLAT

101 SLSMFYLLRI ANFSNLIFLR IKRRVKSVVL VILLGPLLFL VCHLFVINMD

151 ETVWTKEYEG NVTWKIKLRS AMYHSNMTLT MLANFVPLTL TLISFLLLIC

201 SLCKHLKKMQ LHGKGSQDPS TKVHIKALQT VTSFLLLCAI YFLSMIISVC

251 NFGRLEKQPV FMLCQTLGIM YPSFHSFFLI MGSRKLKQTF LSVLCQVTCL

301 VKGQQPSTP

**TAS2R19**

1 MMCFLLIISS ILVVFAFVLG NVANGFIALV NVIDWVNTRK ISSAEQILTA

51 LVVSRIGLLW VMLFLWYATV FNSALYGLEV RIVASNAWAV TNHFSMWLAA

101 SLSIFCLLKI ANFSNLISLH LKKRIKSVVL VILLGPLVFL ICNLAVITMD

151 ERVWTKEYEG NVTWKIKLRN AIHLSSLTVT TLANLIPFTL SLICFLLLIC

201 SLCKHLKKMR LHSKGSQDPS TKVHIKALQT VTSFLMLFAI YFLCIITSTW

251 NLRTQQSKLV LLLCQTVAIM YPSFHSFILI MGSRKLKQTF LSVLWQMTR

**TAS2R20**

1 MMSFLHIVFS ILVVVAFILG NFANGFIALI NFIAWVKRQK ISSADQIIAA

51 LAVSRVGLLW VILLHWYSTV LNPTSSNLKV IIFISNAWAV TNHFSIWLAT

101 SLSIFYLLKI VNFSRLIFHH LKRKAKSVVL VIVLGSLFFL VCQLVMKNTY

151 INVWTEECEG NVTWKIKLRN AMHLSNLTVA MLANLIPFTL TLISFLLLIY

201 SLCKHLKKMQ LHGKGSQDPS TKIHIKALQT VTSFLVLLAI YFLCLIISFW

251 NSKMLPKEIV LMLCQAFGII YPSFHSFILI WGNKTLKQTF LSVLWQVTCW

301 AKGQNQSTP

**TAS2R50**

1 MITFLYIFFS ILIMVLFVLG NFANGFIALV NFIDWVKRKK ISSADQILTA

51 LAVSRIGLLW ALLLNWYLTV LNPAFYSVEL RITSYNAWVV TNHFSMWLAA

101 NLSIFYLLKI ANFSNLLFLH LKRRVRSVIL VILLGTLIFL VCHLLVANMD

151 ESMWAEEYEG NMTGKMKLRN TVHLSYLTVT TLWSFIPFTL SLISFLMLIC

201 SLYKHLKKMQ LHGEGSQDLS TKVHIKALQT LISFLLLCAI FFLFLIVSVW

251 SPRRLRNDPV VMVSKAVGNI YLAFDSFILI WRTKKLKHTF LLILCQIRC

**TAS2R60**

1 MNGDHMVLGS SVTDKKAIIL VTILLLLRLV AIAGNGFITA ALGVEWVLRR

51 MLLPCDKLLV SLGASRFCLQ SVVMGKTIYV FLHPMAFPYN PVLQFLAFQW

101 DFLNAATLWS STWLSVFYCV KIATFTHPVF FWLKHKLSGW LPWMLFSSVG

151 LSSFTTILFF IGNHRMYQNY LRNHLQPWNV TGDSIRSYCE KFYLFPLKMI

201 TWTMPTAVFF ICMILLITSL GRHRKKALLT TSGFREPSVQ AHIKALLALL

251 SFAMLFISYF LSLVFSAAGI FPPLDFKFWV WESVIYLCAA VHPIILLFSN

301 CRLRAVLKSR RSSRCGTP
